# Measuring Online Social Support: Development and Validation of a Short Form for Chinese Adolescents

**DOI:** 10.3390/ijerph192114058

**Published:** 2022-10-28

**Authors:** Ziyao Zhou, Qijin Cheng

**Affiliations:** Department of Social Work, Chinese University of Hong Kong, Shatin, Hong Kong 999077, China

**Keywords:** adolescent, social support, online social support, confirmatory factor analysis, scale validation, cross-validation

## Abstract

Supportive interactions on social media have great potential to benefit adolescents’ development. However, there is no instrument to measure online social support (OSS) in China. The study aimed to develop and validate a Chinese short version of the Online Social Support Scale (OSSS). The original scale was translated into Chinese through multiple forward and backward translation protocols. The calibration sample (*N* = 262) was used to select items and test the reliability, validity, and internal structure of the short form. The cross-validation sample (*N* = 267) was then used to assess measurement invariance by multigroup confirmatory factor analysis and examine criterion validity based on its relationships with life satisfaction, depression, and time on social media. The 20-item Chinese short version of OSSS (OSSS-CS) includes four factors: esteem/emotional support, social companionship, informational support, and instrumental support. Our results suggest that the OSSS-CS has high internal consistency, construct validity, and criterion validity. Furthermore, evidence of partial cross-validity demonstrated invariance of the variance–covariance matrices, factor structure, factor loadings, and factor variance across independent samples. The results also revealed that the original OSSS could be replicated across cultures. Finally, the short form developed in the study can be used as a reliable and valid measure of online social support among the Chinese adolescent population.

## 1. Introduction

In the digital era, the Internet plays an enormous role in people’s daily lives. Social media platforms have become a global means of social interaction for people of all ages, especially among the younger populations. The use of social media in China is particularly ubiquitous and popular [1]. In 2021, China became the world’s largest social media market, attracting 926 million active social media users [2]. Among them, Chinese adolescents account for a relatively high proportion [3]. Social media is the second-most commonly used smartphone application among Chinese adolescents, with an 88.7% share, just after music apps at 95.8% [4]. It is worth noting that popular international social media such as Facebook, Instagram, and Twitter are blocked in China. Chinese users gather on a completely different set of social media channels such as WeChat, QQ, and Weibo [5]. The independent social media ecosystem in China underscores how different it is from its Western counterparts and the value and importance of studying social interactions in China’s mediascape.

With the proliferation of social media, the way in which adolescents interact has changed dramatically. Scholars have taken note of the trend, resulting in a growing body of research on this topic. While some studies have revealed the potential dangers and adverse impacts of social media, such as cyberbullying and Internet addiction [6,7,8], far less research has focused on the positive aspects of social media [9]. With the pervasive use of social media, it has come to represent new and important sources of or vehicles for social support [10]. Social media may fill a need for social belongingness, provide a distraction from various stressors, or enable people to express and receive support via common features, such as being “friended” or “followed” by others, liking a post, or leaving an encouraging comment [11,12]. A Chinese survey involving 5076 adolescents in six provinces showed that more than half (59%) of the adolescents agreed with the statement, “The Internet brings people closer,” compared with 38.3% of adolescents who agreed with the statement, “The Internet makes people more alienated” [13]. Despite the fact that online social relations convey opportunities and risks, research on their positive side is still in the early stages [9].

Social support is fundamental nourishment in a person’s environment and is crucial to many development stages [14]. The positive role of social support on mental and physical health has been widely acknowledged [15]. In childhood and adolescence, social support is a contributing factor to positive development and general health behaviors, a protective factor from psychological distress, and a predictor of well-being in adulthood [16]. In the traditional face-to-face context, the main effect model suggests that social resources have a beneficial effect on mental health, irrespective of whether or not persons are under stress [15]. The social support generated from online space, so-called online social support (OSS), can be defined as the Internet-facilitated receipt of emotional, informational and instrumental assistance, and sense of belonging from friends, family, and others in one’s social networks [17,18]. OSS appears to have promising influence on adolescents’ well-being and operates in ways that are quite similar to in-person social interactions [19]. A systematic review found that Facebook-based social support improved well-being and general physical and mental health. It was also found to reduce mental illness, including depression, anxiety, and loneliness [20]. However, some evidence showed that social support from social media was related to more significant mental health conditions, e.g., a higher likelihood of depression and poorer quality of life [12,21]. The inconsistent findings regarding the relationship between OSS and adolescents’ mental health were partly because of the varied conceptualization and operationalization of OSS [22].

Current measurements of OSS are mainly altered versions of established face-to-face social support measures based on different theoretical conceptualizations of social support. For example, some studies measured perceptions of online support, i.e., the personal interpretation of supportive transactions, such as the Interpersonal Support Evaluation List [23] used by Indian and Grieve [24] and the family subscale of the Multidimensional Scale of Perceived Social Support [25] used by Frison and Eggermont [26]. Some studies measured received social support, i.e., the amount of social support experienced, such as the Inventory of Socially Supportive Behaviors [27] used by Kim [28] and the tone of reactions in Valkenburg et al. [29]’s study. Additionally, Lin et al. [6] used the Social Support Scale [30] to capture OSS from people acquainted only through the Internet, as distinguished from people known in person. There are several newly developed scales specifically for OSS, such as the Online Social Support Scale (OSSS) [9], which is used to measure the frequency of support behaviors, and the Liang OSS Scale [31], which measures social support perceptions. However, these extant measures tend to rely on ad hoc measures, often without strong psychometric evidence, and their varied theoretical foundation has complicated interpretations [9].

Among these scales, the OSSS is the first to have both theoretical roots and strong psychometric support. To capture the multifaceted nature of OSS, Nick and colleagues conducted a thorough literature review of theoretical and empirical attempts to characterize the structure of in-person social support [9]. They found four factors that have consistently arisen in the literature: (1) esteem/emotional support (EE) refers to expressions of acceptance, intimacy, care, like, respect, etc. through verbal and nonverbal cues from others; (2) social companionship (SC) support fosters a sense of belonging through expressions of inclusivity or spending time together; (3) informational support (INF) includes giving advice and feedback, sharing information, perspectives, and knowledge, and providing resources; and (4) instrumental support (INS) means providing financial, material assistance, helping with tasks, and taking responsibility [9]. This classification divided social support according to its function.

Evidence of reliability, convergent validity, and discriminant validity supports the psychometric properties of the OSSS [9]. Its validity and reliability have been proven in college and community samples in the US [9,32]. However, little is known about the psychometric properties of the OSSS in the adolescent population and Eastern cultures. It is possible that the factor structure of the OSSS might vary across ages, as teens can have different social interaction patterns compared with adults. Junior high school students, in particular, are at the early stage of a transitional period and are just beginning to develop their Internet usage patterns and habits. Furthermore, the validation of measures across cultures and languages is an important step in studying human behaviors that are deemed universal. Studying whether this scale is applicable to China’s unique social media ecological system is necessary and helpful to compare the similarities and differences in OSS across societies, cultures, and media platforms.

As a reliable measurement of OSS is relatively scarce in contemporary China, adapting the OSSS to the Chinese context can be a timely effort to facilitate further research on online social relations among Chinese youth. In addition, developing a Chinese version of the OSSS can have significance for further cross-cultural research on adolescent development in the digital era. Although OSSS has high psychometric qualities and has the potential to be broadly used in future studies, one limitation is that it has 40 items. Completing the full scale takes a long time, and this questionnaire may not be practical in a busy setting. Therefore, a short form is needed to make it more feasible and convenient to administer in community and school settings.

In this study, we aimed to provide a validated Chinese short form of the OSSS (OSSS-CS) for use among the adolescent population. First, we translated and adapted the OSSS into Chinese; then we selected items to develop a short version of the OSSS and tested its psychometric properties in the Chinese adolescent population. To rule out the possibility that the factor structure of the OSSS-CS was achieved only through the post hoc selection of statements that met our selection criteria, we administered the OSSS-CS to another sample to test the replicability of the modified model. Finally, previous research suggested that social media use contributed to OSS [9,33], and OSS was correlated with life satisfaction and depression [10,20,26]. Thus, we demonstrated its criterion validity by examining its relationships with social media use, life satisfaction, and depression.

To accomplish these purposes, we considered five hypotheses, as follows:

**Hypothesis** **1** **(internal** **structure).** *The indicators used to measure OSS reveal four factors that fit the data;*

**Hypothesis** **2** **(reliability).** *The scale and subscales show high internal consistency;*

**Hypothesis** **3** **(construct** **validity).** *The indicators of each latent variable have a high shared variance; each latent variable is unique compared with other constructs;*

**Hypothesis** **4** **(measurement** **invariance).** *The scale shows robust invariance across samples;*

**Hypothesis** **5** **(criterion** **validity).** *The dimensions of the OSSS-CS have positive relations with social media use and life satisfaction, and a negative relation with depression.*

## 2. Materials and Methods

### 2.1. Participants

The data were collected by a paper-based questionnaire survey conducted between September 2021 and December 2021. Approval to conduct the studies was obtained from the Survey and Behavioral Research Ethics Committee of the Chinese University of Hong Kong. The participants consisted of two groups. The calibration sample comprised 262 students in their second year of junior high school from one school in Foshan, Guangdong Province. Students in six classes were invited to participate in the study. A total of 312 students participated, with 262 valid responses. Their ages ranged from 12 to 16 years (mean age = 13, SD = 0.56), and 47.7% were female. The cross-validation sample consisted of 267 students in two other junior high schools in Foshan. One class was randomly chosen from each grade of each school. Thus, a total of six classes were involved, and all students in the chosen classes were invited to participate in the survey. A total of 320 students filled out the questionnaire, with 267 valid responses. This sample comprised 137 (51.3%) males and 130 (48.7%) females, whose ages ranged from 12 to 15 years (mean age 13, SD = 1.00). The demographics of the two separate samples are shown in Table 1. A total of 529 middle school students were included in the study. As can be seen, there are more boys than girls among the participants. The average age of the participants was 13 years old, and over half of the students lived on campus. Around 80% of the students’ household registrations were in Foshan, while the rest were from other regions. The majority of students rated their family income as similar to or better than average. In terms of social media use, students spent more time on social media during weekends and holidays than on weekdays.

### 2.2. Measures

The OSSS is a five-point Likert scale comprising 40 items. It has four subscales: esteem/emotional support (e.g., “People encourage me when I’m online”), social companionship (e.g., “When I’m online, I talk or do things with other people”), informational support (e.g., “People help me learn new things when I’m online”), and instrumental support (e.g., “When I’m online, people help me with school or work”). Items inquire about how often the supportive interactions have happened while the participants interacted with others online over the last two months. Each item was scored using a five-point Likert scale, wherein a higher score indicates greater OSS. The scale is well-validated and has a high degree of internal consistency; the coefficient alpha ranged from 0.94 to 0.95 [9].

Depression was measured using the short form of the Center for Epidemiological Studies Depression Scale (CES-D) [34]. The 10-item CES-D demonstrated good reliability and construct validity in the adolescent population [35]. Among Chinese adolescents (aged 11–18), Cronbach’s α was 0.85 China (r = 0.85), which presents a viable alternative to the full instrument [36]. In the CES-D, the participants were asked to consider their past week while answering questions on a scale of 0–3 (0 = rarely or none of the time (less than 1 day), 1 = some or a little of the time (1–2 days), 2 = occasionally or a moderate amount of time (3–4 days), 3 = most or all of the time (5–7 days)). In this study, Cronbach’s α was 0.86.

Life satisfaction was measured with the Satisfaction with Life Scale (SWLS) [37], a five-item self-report instrument intended to assess satisfaction with one’s life as a whole. The respondents indicate their degree of agreement with each statement of the SWLS using a seven-point Likert scale (7 = “strongly agree,” 1 = “strongly disagree”). The SWLS has been extensively used and translated into more than 25 languages, showing good reliability and validity [38]. The instrument has also been shown to have excellent validity and internal consistency (α = 0.76) in the Chinese adolescent population [39]. In the current study, the reliability of the scale was 0.86.

Social media use was measured by asking participants how many hours per day during weekdays (versus weekends and holidays) they spent using social media (e.g., WeChat, QQ, and Weibo).

We also collected some basic information about the participants by self-report. Students were asked to indicate their household registration (whether from Foshan or other areas) and living arrangements (whether living on campus during weekdays) and to rate their family income (as high, middle, or low).

### 2.3. Translation Procedure

Multiple forward- and backward-translation protocol was used in translation. Two bilingual Social Work PhD students independently translated the OSSS into Chinese. Chinese was the native language of all translators, and they possessed a high level of English language proficiency. Inconsistency was solved through discussion. A third PhD student who was blinded to the original version retranslated the Chinese version to English, and the back-translated scale was compared with the original scale. Only a minor inconsistency was identified, which was further resolved by a discussion between the third and the previous two translators. The pretesting of the Chinese version was performed first among six social workers in youth service and then by 10 junior high school students. They were invited to review the translated scale and make suggestions wherever necessary. The clarity of the items, the relevance of the content to online situations, the simplicity of the instructions, and the capacity of teen respondents to complete the scale were all considered in the review. The tool was finalized based on these discussions. The final version of the 40-item Chinese OSSS was conducted on the calibration sample.

### 2.4. Data Analysis

First, descriptive statistics of the OSSS-CS and the four subscales were analyzed. Then, confirmatory factor analysis (CFA) was conducted on the calibration sample with a four-factor structure. To select the items of the original measure that best assessed the construct, we followed the guideline of item selection for the short-form scale development of Marsh et al. [40]. The following criteria were used in this study: (1) reducing the length of the OSSS by 50% (i.e., to retain no more than 20 items), (2) retaining the same content coverage of the four dimensions of the OSSS, (3) retaining at least five items in each subscale, (4) maintaining at least similar alpha coefficients for each subscale, (5) achieving an item-total correlation of at least 0.40, (6) providing a factor structure with acceptable goodness of fit indexes, (7) ensuring that the size of standardized item factor loadings must be CFA > 0.4, and (8) checking whether items have minimum cross-loading and correlated uniqueness.

Once the items of the OSSS-CS were selected, we conducted CFA to test the structural validity of OSSS-CS. The goodness of fit indices include a non-normed fit index (NNFI), a comparative fit index (CFI), a standardized root mean square residual (SRMR), and a root mean square error of approximation (RMSEA). The NNFI and CFI values, ≥0.90 and ≥ 0.95, respectively, were indicative of acceptable and good model fit, as were the corresponding SRMR (≤0.10 and ≤ 0.08) and RMSEA values (≤0.08 and ≤0.06) [41,42]. We then calculated Cronbach’s α to examine the scales’ internal consistency and computed the correlations between the original measure and the shortened version.

Next, to assess the generalizability of the OSSS-CS, we cross-validated the scale using a multistep procedure. We tested loose, partial, and tight cross-validation based on the progression of invariance constraints specified by MacCallum et al. [43]. In loose cross-validation, all the parameters in the model are re-estimated in an independent sample (i.e., the cross-validation sample) [44]. In partial cross-validation, a series of nested models were performed with added constraints on item loadings, factor covariances, and uniqueness values successively. The criteria for tight cross-validation are satisfied when all of the parameters in the model are held equal between samples and are able to reproduce the observed covariances in the second sample [43]. The nonsignificant results of the chi-square difference test (*p* > 0.01) [45], |ΔCFI| < 0.01 [46], and |ΔRMSEA| < 0.015 [47] would support the invariance assumption. Finally, Pearson correlations of the total scale and subscale scores with mental health variables and social media use were assessed to verify criterion validity in the cross-validation sample. Data analyses were performed with SPSS 25.0 (IBM: New York, NY, USA) and Amos 23.0 (IBM: Chicago, IL, USA).

## 3. Results

### 3.1. Descriptive Analyses

Table 2 presents the means and standard deviations of the variables of interest for both the calibration and cross-validation samples. The collected responses suggested that adolescents exhibited a moderate level of OSS, with instrumental support at a lower level compared with the other three types of OSS. Values’ skewness and kurtosis indicated normal univariate distribution in full-scale scores and subscales’ scores. However, multivariate normality was violated (Mardia’s statistic > 5). As such, in the following CFA, we employed ML estimation using 2000 bootstrap samples to calculate the Bollen–Stine adjustment for the chi-square *p* value [48].

### 3.2. Calibration Sample Analyses

Using the proposed criteria, 20 items were selected to construct a short form scale of the OSSS-CS (emotional support 1, 2, 3, 4, and 7; companionship support 11, 12, 13, 14, and 20; informational support 22, 24, 25, 26, and 27; and instrumental support 32, 34, 36, 37, 39. See Appendix A, Table A1). The total score of the OSSS-CS was highly and significantly correlated with the full scale (r = 0.99, *p* < 0.000), and the OSSS-CS explained 76.51% of the variance of the full scale.

Internal consistency reliability was assessed by calculating Cronbach’s alpha. Results from the calibration sample indicated that the coefficient alpha value for the 20-item OSSS-CS was 0.963, while those for esteem/emotional support, social companionship, informational support, and instrumental support were 0.917, 0.8, 98 0.935, and 0.922, respectively, suggesting good internal consistency for the OSSS-CS and for each factor.

Next, CFA was conducted for the short version in the calibration sample. The four-factor model of the OSSS-CS fitted the data well (χ^2^/df = 2.105, *p* = 0.001, Bollen–Stine *p* = 0.066, CFI = 0.961, NFI = 0.928, PGFI = 0.065, RMSEA = 0.065, SRMR = 0.044). All factor loadings were statistically significant and above 0.7 (see Table 3). The composite reliability (CR) indicates that all items of each latent variable constantly measure their corresponding construct. In all cases, CR was higher than 0.90. Thus, the indicators of the four factors are reliable measures of the constructs. Table 4 displays the results of convergent and discriminate validities. Convergent validity is supported if the average variance extracted (AVE) value is greater than 0.50. In this study, the AVE showed values higher than 0.5 in all cases. Thus, a substantial amount of the variance of the indicators is explained by the construct compared with the error of the measurement. Discriminant validity is evident if the square root of every AVE value belonging to each latent construct is much larger than any correlation among any pair of latent constructs [49]. This criterion is fulfilled in the calibration sample.

### 3.3. Cross-Validation Sample Analyses

To test the generality of the adapted short version, reliability and construct validity were examined in the cross-validation sample, and the measurement invariance was assessed across the two samples.

The results from the cross-validation sample showed that the coefficient alpha value for the 20-item OSSS-CS was 0.969, while those for esteem/emotional support, social companionship, informational support, and instrumental support were 0.944, 0.900, 0.932, and 0.913, respectively. Therefore, the results from the cross-validation sample indicated the high internal consistency reliability of the short form of the OSSS-CS. In the cross-validation sample, CR was higher than 0.9, and the AVE value was greater than 0.60. The criteria of composite reliability and convergent validity were fulfilled. Furthermore, the criterion of discriminate validity was achieved in all but two cases. Specifically, the square roots of the AVE of INF (0.859) and INS (0.822) were slightly lower than the correlation between them. Likewise, the square root of the AVE of SC (0.806) was slightly lower than its correlations with INF (0.859) and INS (0.822).

To assess the cross-validity of the short scale adapted from the calibration sample, a series of progressively more restrictive models was tested (Table 5). First, loose cross-validation was performed. This model was specified as described previously and fitted the data well. Then, the equal variance-covariance matrices model was tested on the total sample (combination of calibration and cross-validation samples), and the model fitted well. Factor structure, item loadings, factor variances-covariances, and uniqueness values were sequentially constrained to be equal. The chi-square difference tests between these nested models were statistically significant and sensitive to sample size. Changes in CFI and RMSEA were lower than the thresholds except for uniqueness, suggesting that the factor structure, factor loadings, and factor variances were invariant across the calibration and cross-validation samples.

Criterion validity was estimated by correlating the scores of the OSSS-CS and the four subscales with participants’ mental health (i.e., depression and life satisfaction) and social media use in the cross-validation sample. As shown in Table 6, life satisfaction was positively correlated with the OSSS-CS and the four dimensions, while depression was only negatively related to informational support (r = −0.124, *p* < 0.05) and instrumental support (r = −0.128, *p* < 0.05). Adolescents’ social media use was significantly related to OSS, regardless of whether this occurred on weekdays or weekends/holidays, but the correlation was stronger during the latter.

## 4. Discussion

Social media has become an important part of adolescents’ daily lives, providing a new social context for their interactions [50]. In recent years, the literature has begun to document the positive effects of online social interaction [9]. The accurate measurement of OSS is critical for practitioners and researchers to improve our understanding of OSS and capture its benefits. The OSSS is the first OSS measurement that has both deep theoretical roots and strong psychometric properties [9]. The current study developed a short version of OSSS to be used in the Chinese adolescent population.

The results of the CFA support Hypothesis 1, which states that the OSSS-CS reserved the full scale’s four-factor structure: esteem/emotional support, social companionship, informational support, and instrumental support. The findings demonstrated that the subtypes of social support that apply to the US college and university populations also apply to the Chinese adolescents being studied. The overall OSSS-CS and the four subscales displayed good reliability in the two samples, thus supporting Hypothesis 2. Convergent validity was supported by AVE values, and discriminant validity was confirmed in the calibration sample as the AVE in each factor was greater than the square of this correlation with the other scale factors. However, in the cross-validation sample, the square root of the AVE of SC was slightly lower than its correlation with other latent constructs, suggesting that adolescents may feel companionship from other supportive activities. Thus, it might be difficult to distinguish social companionship support from other dimensions. Future studies may benefit from qualitative studies to reveal the meaning that adolescents attach to OSS.

The measurement invariance hypothesis was partially supported. Although the results demonstrated invariance of the covariance matrices, factor structure, factor loadings, and factor variance across two independent samples, the criteria for tight cross-validation are not satisfied as uniqueness values were not equal between the groups. Tight cross-validation is the strongest evidence for cross-validity, but because the measurement and specific errors inherent in uniqueness values are assumed to be random across samples, it may not be realistic to achieve [43]. Thus, when the item loadings and factor covariances can be constrained to equality across samples, partial cross-validation evidence for a measure is considered quite acceptable.

The criterion validity hypothesis was partly supported. In particular, the results demonstrated a positive correlation between OSSS-CS and life satisfaction. This is in line with previous studies [29,39] and can be explained by the main effect model of social support, which states that social support has a generalized beneficial effect [51]. This finding suggests that in-person social support theory can be extended to the online world of social relations. Moreover, given that the data are cross-sectional, it is also possible that adolescents with higher life satisfaction received more social support on social media. The overall OSS was not significantly associated with depression, while the instrumental and informational dimensions of OSS were negatively associated with depression. A recent meta-analysis revealed a small and insignificant correlation between OSS and adolescents’ depression (r = −0.09) [22]. As previous studies mainly discussed the overall impact of OSS, the findings of our study highlight the need to further examine the effects of the dimensions of OSS and the individual difference characteristics that moderate such effects. In line with Nick et al. [9], the results of the current study showed that spending more time on social media was associated with a higher level of OSS. The study further revealed that all kinds of OSS were increased with more time spent on social media.

The current study contributes to the current literature in several ways. First, to the best of our knowledge, this is the first psychometrically sound instrument in Chinese to measure OSS received from social media. The study expanded the utility of the OSSS to the Chinese context. The scale can serve as an important tool for assessing differences and similarities in OSS across social, cultural, and media contexts. Second, our study demonstrated the replicability of the OSSS in the adolescent population (aged 12−16) by demonstrating its reliability and validity. Third, the study improved the brevity and ease of administration of the original scale by reducing the form from 40 to 20 items. Overall, the present study highlights the value of the OSSS in measuring OSS. The tool is now readily available for future research on OSS among the Chinese adolescent population and its corresponding antecedents and consequences.

Although the study provides a useful scale for researchers, some limitations must be considered. First, convenience sampling at the school level limits the generalizability to other schools. Thus, future research must use probability sampling strategies to improve representativeness. Second, the present study has a narrowed focus on junior high school students, as early adolescence is a critical period for educating young people on how to use the Internet and media. The OSS-CS’s utility in the Chinese university student sample and the general sample has not yet been examined. Thus, future studies may benefit from extending the investigation to other populations in China and replicating the study in diverse cultural contexts. Third, the study only assessed the association of the OSSS-CS with depression and life satisfaction. Future research can examine the relationship of the OSSS-CS to a wider variety of outcomes, which would help verify the criterion validity of the measure and fully understand the role OSS played in adolescents’ well-being. Finally, the present study indicated the general benefits of OSS; however, the group differences and mechanisms underlying its benefits remain unknown. Furthermore, longitudinal studies are required to identify predictors of the OSSS-CS.

## 5. Conclusions

This study adapted and validated a short measure of the OSSS and confirmed that it includes four factors, i.e., esteem/emotional support, social companionship, informational support, and instrumental support. Our results suggest that the OSSS-CS has high internal consistency, construct validity, and criterion validity and demonstrated the invariance of the variance-covariance matrices, factor structure, factor loadings, and factor variance across two independent samples. The OSSS-CS can serve as a useful tool in assessing the online occurrence of social support among the Chinese adolescent population.

## Figures and Tables

**Table 1:** General characteristics of participants.

		*N*		%	
Gender	Male	137	*137*	52.3	*51.3*
	Female	125	*130*	47.7	*48.7*
Grade	1		*99*		*37.1*
	2	262	*80*	100.0	*30.0*
	3		*88*		*33.0*
Living arrangement	Residential	155	*178*	59.2	*66.7*
	Commuter	107	*89*	40.8	*33.3*
Household registration	Local	216	*209*	82.4	*78.3*
	Non-local	46	*58*	17.5	*21.8*
Family income	High	146	*156*	55.7	*58.4*
	Middle	101	*96*	38.5	*36.0*
	Low	15	*15*	5.8	*5.6*
		M (hour)		SD	
Daily time spent on social media	Weekdays	0.17	*0.25*	0.44	*0.56*
	Weekends and holiday	1.78	*1.56*	2.94	*2.09*

Note: Calibration sample in regular font, cross-validation sample in italics.

**Table 2:** Descriptive statistics for the OSSS-CS across the entire sample.

	M		SD		Skewness		Kurtosis	
OSSS-CS	2.71	*2.91*	0.82	*1.06*	0.36	*−0.06*	0.05	*−0.74*
EE	2.85	*2.86*	0.89	*1.16*	0.06	*0.09*	−0.06	*−0.81*
SC	2.76	*3.00*	0.97	*1.17*	0.21	*−0.05*	−0.28	*−0.94*
INF	2.79	*2.99*	0.95	*1.18*	0.25	*−0.02*	−0.09	*−0.92*
INS	2.43	*2.77*	0.96	*1.14*	0.62	*0.14*	0.26	*−0.75*

Note: Calibration sample in regular font, cross-validation sample in italics. Abbreviations: OSSS-CS, online social support scale-Chinese short version; EE, emotional/esteem support; SC, social companionship support; INF, informational support; INS, instrumental support.

**Table 3:** Results of CFA among calibration sample.

Factors	Indicators	Est.	SE.	*p*	Factor Loading	SMC	CR	AVE
EE	Item 1	1			0.91	0.827	0.921	0.704
	Item 2	0.948	0.039	***	0.916	0.839		
	Item 3	1.013	0.040	***	0.927	0.86		
	Item 4	0.748	0.052	***	0.713	0.509		
	Item 7	0.763	0.056	***	0.691	0.478		
SC	Item 11	1			0.752	0.565	0.901	0.646
	Item 12	1.214	0.079	***	0.903	0.815		
	Item 13	1.218	0.082	***	0.882	0.778		
	Item 14	1.020	0.085	***	0.732	0.535		
	Item 20	1.036	0.086	***	0.733	0.537		
INF	Item 22	1			0.82	0.672	0.934	0.742
	Item 24	1.108	0.060	***	0.907	0.823		
	Item 25	1.054	0.065	***	0.835	0.698		
	Item 26	1.043	0.058	***	0.893	0.798		
	Item 27	1.035	0.062	***	0.849	0.721		
INS	Item 32	1			0.765	0.585	0.923	0.707
	Item 34	1.128	0.076	***	0.848	0.719		
	Item 36	1.216	0.075	***	0.91	0.829		
	Item 37	1.119	0.078	***	0.83	0.688		
	Item 39	1.111	0.075	***	0.844	0.713		

Abbreviations: Est. = estimation, SE. = standard error, SMC = squared multiple correlations, CR = construct reliability, AVE = average variance extracted; *** *p* < 0.001; EE, emotional/esteem support; SC, social companionship support; INF, informational support; INS, instrumental support.

**Table 4:** Results of the convergent and discriminant validity of the OSSS-CS.

	AVE	EE	SC	INF	INS
EE	0.704	0.839			
SC	0.646	0.664	0.804		
INF	0.742	0.727	0.734	0.861	
INS	0.707	0.662	0.715	0.831	0.841

Abbreviations: AVE, average variance extracted; EE, emotional/esteem support; SC, social companionship support; INF, informational support; INS, instrumental support.

**Table 5:** Fit Indexes for cross-validation and invariance analyses.

Model	df	χ^2^	RMSEA	CFI	SRMR
Cross-validation sample	164	402.226	0.074	0.953	0.040
Equivalent covariance matrices	164	412.617	0.054	0.974	0.033
Equivalent factor structure	328	747.514	0.049	0.957	0.044
Fixed structure and item loading					
	344	791.287	0.050	0.954	0.042
Fixed structure, item loading, and factor covariance					
	354	823.343	0.050	0.951	0.077
Fixed structure, item loading, factor variance/covariance, and uniqueness					
	374	1098.811	0.061	0.925	0.059
**Model Comparison**	**Δdf**	**Δχ^2^**	**ΔRMSEA**	**ΔCFI**	***p***
Item loadings-structure	16	43.773	0.001	0.003	0.000
Factor variances/covariances-item loadings	10	32.056	0.000	0.003	0.000
Uniqueness- Factor variance/covariance	20	275.468	0.011	0.026	0.000

Abbreviations: RMSEA, root mean square error of approximation; SRMR, standardized root mean square residual; CFI, comparative fit index.

**Table 6:** Results of criterion validity among the cross-validation sample.

		OSSS	EE	SC	INF	INS
Depression		–0.111	–0.087	–0.065	–0.124 *	–0.128 *
Life satisfaction		0.283 **	0.255 **	0.226 **	0.277 **	0.267 **
Social media use	Weekdays	0.136 *	0.098	0.145 *	0.119	0.132 *
	Weekend & holiday	0.173 **	0.140 *	0.149 *	0.153 *	0.186 **

* *p* < 0.05, ** *p* < 0.01. Abbreviations: OSSS, online social support scale; EE, emotional/esteem support; SC, social companionship support; INF, informational support; INS, instrumental support.

## Data Availability

The datasets generated during and/or analyzed during the current study are not publicly available due to datasets containing information that could compromise the privacy of research participants. The data that support the findings of this study are available from the corresponding author upon reasonable request.

## Appendix A

**Table A1:** Online Social Support Scale—Chinese Short Version.

	1 = 从未发生 2 = 很少发生 3 = 有时发生 4 = 经常发生 5 = 总是发生 [1 = Never 2 = Rarely 3 = Sometimes 4 = Often 5 = A lot]					
1.	在社交媒体上, 有人关心我 [People show that they care about me online.]	1	2	3	4	5
2.	在社交媒体上, 有人说或做的事情让我对自己感觉良好 [Online, people say or do things that make me feel good about myself.]	1	2	3	4	5
3.	在社交媒体上, 有人鼓励我 [People encourage me when I’m online.]	1	2	3	4	5
4.	在社交媒体上, 有人关注我 [People pay attention to me online.]	1	2	3	4	5
5.	在社交媒体上, 有人告诉我他们欣赏我说的话或我做的事情 [When I’m online, people tell me they like the things I say or do.]	1	2	3	4	5
6.	在社交媒体上, 我和别人聊天或做事情 [When I’m online, I talk or do things with other people.]	1	2	3	4	5
7.	有人在社交媒体上花时间陪我 [People spend time with me online.]	1	2	3	4	5
8.	有人在社交媒体上和我一起做有趣的事情 [People hang out and do fun things with me online.]	1	2	3	4	5
9.	在社交媒体上, 我在一个志趣相投的团体中 [Online, I belong to groups of people with similar interests.]	1	2	3	4	5
10.	在社交媒体上, 有人让我有归属感 [Online, people make me feel like I belong.]	1	2	3	4	5
11.	在社交媒体上, 有人提供给我有用的信息 [Online, people provide me with helpful information.]	1	2	3	4	5
12.	在社交媒体上, 在我需要时, 有人告诉我去哪寻求帮助 [Online, people would tell me where to find help if I needed it.]	1	2	3	4	5
13.	在社交媒体上有人帮我学习新的东西 [People help me learn new things when I’m online.]	1	2	3	4	5
14.	有人在社交媒体上给我提供建议 [People offer suggestions to me online.]	1	2	3	4	5
15.	在社交媒体上, 有人告诉我我想知道的事情 [People tell me things I want to know online.]	1	2	3	4	5
16.	当我在社交媒体上, 有人帮助我的学习或帮助我完成任务 [When I’m online, people help me with school or work.]	1	2	3	4	5
17.	如果我需要帮助, 我在社交媒体上寻找可以帮助我的人 [If I needed a hand doing something, I go online to find people who will help out.]	1	2	3	4	5
18.	在社交媒体上, 有人帮我解决重要的事情 [Online, people help me with causes or events that I think are important.]	1	2	3	4	5
19.	在社交媒体上, 有人提供我需要的东西 [When I’m online, people have offered me things I need.]	1	2	3	4	5
20.	当我的学习或任务需要帮助, 我在社交媒体上得到他人的帮助 [When I need a hand with school or work things, I get help from others online.]	1	2	3	4	5

## References

[1] Liu C., Ma J. (2018). Social support through online social networking sites and addiction among college students: The mediating roles of fear of missing out and problematic smartphone use. Curr. Psychol..

[2] Statista Number of Social Network Users in Selected Countries in 2021 and 2025. https://www.statista.com/statistics/278341/number-of-social-network-users-in-selected%20countries/.

[3] CNNIC Research Report on the Internet Usage of National Minors in 2020. http://www.cnnic.net.cn/hlwfzyj/hlwxzbg/qsnbg/202107/P020210720571098696248.pdf.

[4] Ji W., Shen J. Annual Report on the Internet Use of Chinese Minors. https://www.pishu.com.cn/skwx_ps/bookdetail?SiteID=14&ID=11860761#.

[5] Statista Social Networks in China—Statistics & Facts. https://www.statista.com/topics/1170/social-networks-in-china/#topicHeader__wrapper.

[6] Lin M.-P., Wu J.Y.-W., You J., Chang K.-M., Hu W.-H., Xu S. (2018). Association between online and offline social support and internet addiction in a representative sample of senior high school students in Taiwan: The mediating role of self-esteem. Comput. Hum. Behav..

[7] Mazzoni E., Baiocco L., Cannata D., Dimas I. (2016). Is internet the cherry on top or a crutch? Offline social support as moderator of the outcomes of online social support on Problematic Internet Use. Comput. Hum. Behav..

[8] Wang L., Ngai S.S. (2021). Understanding the effects of personal factors and situational factors for adolescent cyberbullying perpetration: The roles of internal states and parental mediation. J. Adolesc..

[9] Nick E.A., Cole D.A., Cho S.-J., Smith D.K., Carter T.G., Zelkowitz R.L. (2018). The Online Social Support Scale: Measure development and validation. Psychol. Assess..

[10] Cole D.A., Nick E.A., Zelkowitz R.L., Roeder K.M., Spinelli T. (2017). Online social support for young people: Does it recapitulate in-person social support; can it help?. Comput. Hum. Behav..

[11] Bayer J.B., Triệu P., Ellison N.B. (2020). Social Media Elements, Ecologies, and Effects. Annu. Rev. Psychol..

[12] Shensa A., Sidani J.E., Escobar-Viera C.G., Switzer G.E., Primack B.A., Choukas-Bradley S. (2020). Emotional support from social media and face-to-face relationships: Associations with depression risk among young adults. J. Affect. Disord..

[13] Ban J. (2018). Meijie Shidai de Xuesheng Gongmin Suyang Jiqi Peiyu [Cultivating the Students’ Civic Literacy in Media Age].

[14] Rueger S.Y., Malecki C.K., Pyun Y., Aycock C., Coyle S. (2016). A meta-analytic review of the association between perceived social support and depression in childhood and adolescence. Psychol. Bull..

[15] Cohen S., Underwood L.G., Gottlieb B.H. (2000). Social Support Measurement and Intervention: A Guide for Health and Social Scientists.

[16] Wu Y.-J., Outley C., Matarrita-Cascante D., Murphrey T. (2015). A Systematic Review of Recent Research on Adolescent Social Connectedness and Mental Health with Internet Technology Use. Adolesc. Res. Rev..

[17] Zhao C., Xu H., Lai X., Yang X., Tu X., Ding N., Lv Y., Zhang G. (2021). Effects of Online Social Support and Perceived Social Support on the Relationship Between Perceived Stress and Problematic Smartphone Usage Among Chinese Undergraduates. Psychol. Res. Behav. Manag..

[18] House J.S. (1981). Work Stress and Social Support.

[19] Goncalves B., Perra N., Vespignani A. (2011). Modeling Users’ Activity on Twitter Networks: Validation of Dunbar’s Number. PLoS ONE.

[20] Gilmour J., Machin T., Brownlow C., Jeffries C. (2020). Facebook-based social support and health: A systematic review. Psychol. Popul. Media.

[21] McCloskey W., Iwanicki S., Lauterbach D., Giammittorio D.M., Maxwell K. (2015). Are Facebook “Friends” Helpful? Development of a Facebook-Based Measure of Social Support and Examination of Relationships Among Depression, Quality of Life, and Social Support. Cyberpsychology Behav. Soc. Netw..

[22] Zhou Z., Cheng Q. (2022). Relationship between online social support and adolescents’ mental health: A systematic review and meta-analysis. J Adolesc..

[23] Cohen S., Hoberman H.M. (1983). Positive events and social supports as buffers of life change stress. J. Appl. Soc. Psychol..

[24] Indian M., Grieve R. (2014). When Facebook is easier than face-to-face: Social support derived from Facebook in socially anxious individuals. Pers. Individ. Differ..

[25] Zimet G.D., Dahlem N.W., Zimet S.G., Farley G.K. (1988). The Multidimensional Scale of Perceived Social Support. J. Personal. Assess..

[26] Frison E., Eggermont S. (2015). The impact of daily stress on adolescents’ depressed mood: The role of social support seeking through Facebook. Comput. Hum. Behav..

[27] Barrera M., Sandler I.N., Ramsay T.B. (1981). Preliminary development of a scale of social support: Studies on college students. Am. J. Community Psychol..

[28] Kim H. (2014). Enacted social support on social media and subjective well-being. Int. J. Commun..

[29] Valkenburg P.M., Peter J., Schouten A. (2006). Friend Networking Sites and Their Relationship to Adolescents’ Well-Being and Social Self-Esteem. Cyber. Psychol. Behav..

[30] Yeh Y.-C., Ko H.-C., Wu J.Y.-W., Cheng C.-P. (2008). Gender Differences in Relationships of Actual and Virtual Social Support to Internet Addiction Mediated through Depressive Symptoms among College Students in Taiwan. Cyber. Psychol. Behav..

[31] Liang X., Liu H. (2010). Qingshaonian wangluo shehui zhichi de jiegouxing tansuo [Exploration of the structure of youth online social support]. Jiaoyu Yanjiu Yu Shiyan.

[32] Rabasco A., Corcoran V., Andover M. (2021). Alone but not lonely: The relationship between COVID-19 social factors, loneliness, depression, and suicidal ideation. PLoS ONE.

[33] Liu C.-Y., Yu C.-P. (2013). Can Facebook Use Induce Well-Being?. Cyberpsychology Behav. Soc. Netw..

[34] Andresen E.M., Malmgren J.A., Carter W.B., Patrick D.L. (1994). Screening for Depression in Well Older Adults: Evaluation of a Short Form of the CES-D. Am. J. Prev. Med..

[35] Bradley K.L., Bagnell A.L., Brannen C.L. (2010). Factorial Validity of the Center for Epidemiological Studies Depression 10 in Adolescents. Issues Ment. Health Nurs..

[36] Yang W., Xiong G., Garrido L.E., Zhang J.X., Wang M.-C., Wang C. (2018). Factor structure and criterion validity across the full scale and ten short forms of the CES-D among Chinese adolescents. Psychol. Assess..

[37] Diener E., Emmons R.A., Larsen R.J., Griffin S. (1985). The Satisfaction With Life Scale. J. Pers. Assess..

[38] Pavot W., Michalos A.C. (2014). Satisfaction with Life Scale (SWLS), an Overview. Encyclopedia of Quality of Life and Well-Being Research.

[39] Liu Q., Sun X., Zhou Z., Niu G., Kong F., Lian S. (2016). The Effect of Honest Self-Presentation in Online Social Network Sites on Life Satisfaction: The Chain Mediating Role of Online Positive Feedback and General Self-Concept. J. Psychol. Sci..

[40] Marsh H.W., Ellis L.A., Parada R.H., Richards G., Heubeck B.G. (2005). A Short Version of the Self Description Questionnaire II: Operationalizing Criteria for Short-Form Evaluation With New Applications of Confirmatory Factor Analyses. Psychol. Assess..

[41] Hu L.T., Bentler P.M. (1999). Cutoff criteria for fit indexes in covariance structure analysis: Conventional criteria versus new alternatives. Struct. Equ. Model. Multidiscip. J..

[42] Browne M.W., Cudeck R. (1992). Alternative ways of assessing model fit. Sociol. Methods Res..

[43] MacCallum R.C., Roznowski M., Mar C.M., Reith J.V. (1994). Alternative Strategies for Cross-Validation of Covariance Structure Models. Multivar. Behav. Res..

[44] Bentler P.M., Bonett D.G. (1980). Significance tests and goodness of fit in the analysis of covariance structures. Psychol. Bull..

[45] Kim E.S., Cao C., Wang Y., Nguyen D.T. (2017). Measurement Invariance Testing with Many Groups: A Comparison of Five Approaches. Struct. Equ. Model. A Multidiscip. J..

[46] Cheung G.W., Rensvold R.B. (2002). Evaluating Goodness-of-Fit Indexes for Testing Measurement Invariance. Struct. Equ. Model. Multidiscip. J..

[47] Chen F.F. (2007). Sensitivity of Goodness of Fit Indexes to Lack of Measurement Invariance. Struct. Equ. Model. A Multidiscip. J..

[48] Bollen K.A., Stine R.A. (1992). Bootstrapping Goodness-of-Fit Measures in Structural Equation Models. Sociol. Methods Res..

[49] Zait A., Bertea P.S.P.E. (2011). Methods for testing discriminant validity. Manag. Mark. J..

[50] Wang W., Qian G., Wang X., Lei L., Hu Q., Chen J., Jiang S. (2019). Mobile social media use and self-identity among Chinese adolescents: The mediating effect of friendship quality and the moderating role of gender. Curr. Psychol..

[51] Cohen S., Wills T.A. (1985). Stress, social support, and the buffering hypothesis. Psychol. Bull..

